# Privately made firearm in the US: results from a national survey

**DOI:** 10.1186/s40621-026-00661-w

**Published:** 2026-02-22

**Authors:** Symphony Fletcher, Deborah Azrael, Matthew Miller

**Affiliations:** 1 Harvard T. H. Chan School of Public Health, Boston, MA USA; 2 University of Chicago, Chicago, IL USA; 3 Bouve College of Health Sciences, Northeastern University, Boston, MA USA

## Abstract

**Background:**

Almost nothing is known about the size or composition of the stock of privately made firearms (PMF).

**Objective:**

Estimate the size and composition of the U.S. PMF stock.

**Methods:**

A probability-based online survey of US adults conducted in December 2024 were used to generate nationally representative estimates. 12,907/ 20,907 adults (59%) responded to the survey invitation; 4,059 owned a working firearm. Respondents were asked whether they owned PMFs, defined as “firearms that were NOT produced by a manufacturer (sometimes called privately made firearms), such as guns that were assembled from a kit, 3-D printed or otherwise put together” and if so, how many and what types. They were also asked about their most recent PMF acquisition.

**Results:**

We estimate that 2.9 million [CI: 2.2 million, 3.8 million] US adults own approximately 10.1 million PMFs. Most were constructed from kits (34%) or unfinished parts (32%); 16% were 3D printed, and the remainder were machined from raw materials (18%). Half 49% [CI: 0.43, 0.53] were unserialized. Among US adults who last acquired a PMF within 24 months of the survey, 67.2%, [CI: 48.7, 81.6] had a background check for their most recent acquisition, compared with 45.2% [33.9, 56.9] for PMF owners who last acquired a PMF > 24 months prior (*P* < 0.05). Comparable statistics for serialization were 68.9% [CI: 51.7, 82.1] and 53.8% [42.2, 64.9] (*P* = 0.14).

**Conclusion:**

Approximately half of the 10 million PMFs owned by US adults were not serialized. More recently acquired PMFs are more likely to be serialized.

## Introduction

A privately manufactured firearm (PMF) is a firearm, including a frame or receiver, completed, assembled, or otherwise produced by a person other than a licensed manufacturer. PMFs are assembled using unfinished frames or receivers, partially complete parts kits, 3-D printers, or computerized numerical control CNC milling machines [1]. Some PMFs are referred to as “ghost guns” due to the fact that they cannot be traced by law enforcement, most often due to lack of serialization at time of purchase or production.

From 2017 to 2023, the number of PMFs recovered at crime scenes increased more than 16-fold, from 1,629 to 27,490, suggesting that PMFs may have become increasingly available or desirable for use in crime. Little else is known about size and composition of the stock of PMFs from which these seized PMFs are drawn, whether recently acquired PMFs differ from those acquired more remotely (e.g., by type, serialization), or whether background checks have become a more or less common feature of the PMF acquisition process [2, 3]. 

To our knowledge, the current study is the first nationally representative survey of US adults to (a) estimate the number and types of PMF owned by US adults, (b) characterize the US adults who own these firearms. We also sought to assess serialization and background checks before and after ATF Final Rule 2021R-05 F. [4] That rule, implemented in late August of 2022, required Federal Firearm Licensees (FFLs) to treat PMFs as firearms (and thus to serialize them) and manufacturers of parts and kits to serialize these components.

## Methods

### Sample

Data for this study come from the 2024 National Firearms Survey NFS, a nationally representative web- based survey designed by the authors (DA, MM) and conducted in December 2024 by the research firm Ipsos. Respondents were drawn from Ipsos’ KnowledgePanel KP, an online panel that includes approximately 60,000 U.S. adults. The KP panel is selected on an ongoing basis. Using an equal probability of selection design to provide samples that are representative of the U.S. population. Prior to selection of a specific study sample, Ipsos adjusts panel base weights to account for any discrepancies between panel composition and the distribution of key demographic characteristics of the U.S. population as reflected in the most recent Current Population Survey [5].

For this survey, 21,907 KP members were selected and received an invitation to participate in either English or Spanish, of whom 12,909 (59%) completed the survey. Invitations to participate were sent by email. One reminder email was sent to non-responders three days after initial outreach. Participants were not given any specific incentive to complete this survey, although Ipsos provides raffles and sweepstakes with cash rewards and other prizes for KP participants. The current study sample comprises the 4,059 (28.6%) of these 12,909 respondents who reported that they personally owned a gun.

### Measures

Privately made firearm owners were identified through two questions. First, all respondents were asked “Do you personally own a working gun?” Working guns were defined as “guns that are in working order and capable of being fired”. If participants responded affirmatively, they were asked a series of questions about the number and types of firearms they owned, and then prompted to respond to the question, “Are any of the handguns, long guns or other guns you described above guns that were NOT made by a licensed manufacturer such as Beretta or Bushmaster? Sometimes this type of gun is referred to as a “privately made firearm” or PMF. That is, are any of your guns assembled from a kit, 3-D printed, or otherwise put together from parts?” Respondents were then asked about the number of PMFs they owned, by type (handgun and/or long gun). Respondents who owned PMFs were asked about their most recent PMF acquisition, including when the acquisition occurred, the manufacturing method used, whether or not the PMF was serialized, and the PMF’s cost. Respondents were also asked whether they had undergone a background check at the time they acquired their most recent PMF.

### Statistical analysis

Study weights provided by Ipsos combined pre- sample weights with two study-specific post-stratification weights accounting for oversampling and for survey nonresponse. We report weighted percentages, confidence intervals, and unadjusted odds ratios (OR) using the svy suite of commands in StataNow 18, following the STROBE Strengthening the Reporting of Observational Studies in Epidemiology guidelines for reporting [6]. A single respondent reported owning a total of 100 PMFs and was considered an outlier and thus excluded from estimates of the average number of PMFs owned, but was retained for all other analyses (e.g., number of PMF owners, whether the respondents most recently acquired PMF was serialized).

This work was supported by the Robert Wood Johnson Foundation, the Joyce Foundation and the New Venture Fund. Funders did not play a role in the design, conduct, or reporting of the research, or in the decision to submit the manuscript for publication.

The Harvard School of Public Health and Northeastern University Institutional Review Boards each determined that studies using the NFS meet the criteria for exemption per regulations governing research involving human subjects. The public was not involved in the design or analysis of this research.

## Results

We estimate that 28.6% (*n* = 4059) of US adults owned a firearm in December 2024, of whom, 3.8% [CI: 2.9, 4.0] (*n* = 138) owned at least one PMF (Table 1). Scaling our findings to the U.S. population of adults 18 years and older (266.3 million), [7] we estimate that there are approximately 76.2 million gun owners of whom roughly 2.9 million own at least one PMF (CI: 2.2 million, 3.8 million). Given that the average number of PMFs owned is 3.5 [CI: 1.8, 5.2], we estimate that there are approximately 10.1 million PMFs currently owned by US adults. Estimates including the single outlier who reported owning 100 PMFs yield an estimate of the PMF stock of 12.8 million (not shown).

**Table 1:** Characteristics of owners of privately made firearms and of firearm owners who do not own privately made firearms

	PMF owners (*n* = 138)*	Firearm owners who do not own PMFs (*n* = 3921) weighted column %, 95% CI
**Race**		
White	64.9 [54.9, 73.8]	73.3 [71.7, 74.9]
Black	9.2 [4.9, 16.6]	10.7 [9.6,11.9]
Hispanic	19.5 [12.1, 29.9]	10.5 [9.3,11.8]
Mixed Race	2.4 [1.1, 5.3]	2.0 [1.7, 2.3]
Other	4.0 [1.5, 10.1]	3.5 [2.8, 4.4]
**Age**		
18–20	2.9 [0.7, 10.9]	2.3 [1.7, 3.2]
21–29	12.4 [6.5, 22.5]	9.4 [8.2, 10.8]
30–44	35.7 [27.4, 45.1]	25.5 [23.9, 27.1]
45–59	26.9 [19.6, 35.7]	26.4 [24.9, 27.9]
60+	22.0 [16.0, 29.5]	36.4 [34.8, 37.9]
**Income**		
< 25,000	16.8 [10.8, 25.1]	6.7 [5.9, 7.7]
25,000–74,999	14.8 [9.4, 22.5]	25.0 [23.6, 26.6]
75,000–149,999	32.8 [24.7, 42.1]	37.7 [35.9, 39.3]
> 150,000	35.6 [27.2, 45.0]	30.6 [29.0, 32.3]
**Education**		
Less than high school	11.1 [5.8, 20.4]	5.7 [4.7, 6.8]
High school	27.1 [19.2, 36.7]	27.0 [25.4, 28.7]
Some college	25.1 [18.1, 33.6]	32.1 [30.6, 33.8]
Bachelor’s degree or higher	36.7 [28.6, 45.7]	35.2 [33.6, 36.8]
**Gender**		
Male	64.6 [54.9, 73.3]	65.6 [63.9, 67.2]
Female	35.4 [26.7, 45.1]	34.4 [32.8, 36.1]
**Urbanization**		
Urban	35.5 [27.0,45.0]	30.7 [29.1, 32.4]
Rural	26.7 [1.9, 35.4]	29.5 [27.9, 31.1]
Suburban	37.8 [29.2, 47.2]	39.8 [38.1, 41.5]
**Region**		
Northeast	11.2 [6.4, 18.9]	11.7 [10.6, 12.8]
Midwest	21.5 [14.7, 30.4]	23.0 [21.6, 24.5]
South	41.9 [33.1, 51.2]	44.6 [42.9, 46.3]
West	25.4 [18.1, 34.4]	20.7 [19.3, 22.2]
**Employment Status**		
Full time	67.4 [58.2, 75.3]	56.7 [54.9, 58.4]
Part time	7.2 [3.6, 14.0]	9.8 [8.8, 10.9]
Unemployed	25.4 [18.4, 34.1]	33.5 [31.9, 35.1]
**Veteran Status**		
Yes	30.1 [22.0, 39.7]	16.1 [14.9, 17.3]
No	69.9 [60.3, 78.0]	83.9 [82.7, 85.0]
**Has carried a loaded gun within the past 30 days**		
Yes	57.4 [48.1, 66.1]	25.7 [24.2, 27.3]
No	42.6 [33.9, 51.9]	74.3 [72.7, 75.8]
Types of firearms owned*		
Handgun Only	27.9 [18.4, 39.9]	36.9 [35.0, 38.7]
Long Gun Only	6.4 [2.7, 14.4]	14.3 [13.1, 15.7]
Handgun and Long Gun	65.7 [53.7, 75.9]	48.8 [46.9, 50.7]
**Number of firearms owned**		
1	10.9 [6.3, 18.2]	30.5 [28.9, 32.1]
2	9.4 [5.2, 16.6]	19.3 [17.9, 20.8]
3–5	25.0 [17.5, 34.4]	26.2 [24.7, 27.8]
>=6	54.7 [45.1, 63.9]	24.0 [22.6, 25.5]

*The 138 PMF owners represent 3.8% of all firearm owners [CI: 2.9, 4.0], equivalent to 2.9 million US adults (CI: 2.2 million, 3.8 million)

### Owners of PMFs compared with gun owners without PMFs

Compared with gun owners who did not own PMFs, PMF owners were more likely to be Hispanic 20% [CI: 12.1, 29.9] vs. 11% [CI: 9.3,11.8], to have served in the armed forces 30% [CI: 22.0, 39.7] vs. 16% [CI: 14.9, 17.3] and less likely to be over the age of 60 (22% [CI: 16.0, 29.5] vs. 36% [CI: 34.8, 37.9]). In terms of firearm-related behaviors, PMF owners were more likely than gun owners who did not own PMFs to report having carried a loaded firearm in the last 30 days 57% [CI: 48.1, 66.1] vs. 26% [CI: 24.2, 27.3]. Owners of PMFs also owned more guns than did gun owners who did not own PMFs, for example, 55% [CI: 45.1, 63.9] of PMF owners owned more than 6 total firearms, compared with 24% [CI: 22.6, 25.5] of gun owners who did not own PMFs.

### Characteristics of the stock of PMFs

Most PMFs were assembled from kits 3.4 million (CI: 2.9, 3.9) or unfinished parts (i.e. receivers or frames) 3.2 million [CI: 2.7, 3.6] (Table 2). Approximately half of the PMF stock were handguns (4.7 million) [CI: 4.1, 5.1], and approximately half were unserialized (4.9 million), [CI: 4.3, 5.4].

**Table 2:** Characteristics of the stock of privately made firearms owned by US Adults, December 2024

	Estimated Number of PMFs (95% CI), Millions+
**PMF Type**	
Handgun (*n* = 171)	4.7 (4.1, 5.1)
Long gun (197)	5.4 (4.8, 5.9)
Total (368)	
**Serialization**	
Unserialized (*n* = 178)	4.9 (4.3, 5.4)
Serialized (*n* = 189)	5.2 (4.6, 5.7)
**PMF Construction Method**	
Assembled from kit (*n* = 123)	3.4 (2.9, 3.9)
Printed on 3D printer (*n* = 60)	1.6 (1.3, 2.1)
Machined from raw materials (*n* = 41)	1.1 (0.8, 1.5)
Purchased as an unfinished receiver/frame (*n* = 115)	3.2 (2.7, 3.6)
Other (*n* = 26)	0.7 (0.4, 1.0)
**Region**	
Northeast (*n* = 37)	1.0 (0.7, 1.4)
Midwest (*n* = 106)	2.9 (2.4, 3.4)
South (*n* = 163)	4.5 (3.9, 4.9)
West (*n *= 62)	17 (1.3, 2.1)

+These calculations omit the single respondent who reported owning 100 PMFs. This respondent reported that 50 of his PMFs were handguns and another 50 were long guns, and that all of his PMFs were serialized

### Characteristics of most recently acquired PMFs by PMF acquisition period

Approximately 29% of PMF owners reported having acquired their most recent PMF within the 2 years prior to the survey (Table 3). Among US adults who last acquired a PMF within 24 months of the survey, 67.2%, [CI: 48.7, 81.6] underwent a background check for that acquisition, compared with 45.2% [33.9, 56.9] for PMF owners whose most recent PMF was acquired more than 24 months prior. Comparable statistics for serialization were 68.9% [CI: 51.7, 82.1] and 53.8% [CI: 42.2, 64.9]. The cost, by contrast did not appear to appreciably change, with most PMFs ranging from no cost to less than $400 in both periods.

**Table 3:** Characteristics most recently acquired privately made firearm by recency of last PMF acquisition (within the past 24 months vs. 25 or more months ago)

	Last acquisition within the Past 24 months (*n* = 40)	Weighted Percentage [95% ci]	Last acquisition 25 or more months ago (*n* = 91)	Weighted Percentage [95% ci]	*P*-Value
**Serialization**					0.139
Yes	25	68.9 [51.7, 82.1]	44	53.8 [42.2, 64.9]	
**Background Check**					0.046
Yes	25	67.2 [48.7, 81.6]	36	45.2 [33.9, 56.9]	
**Cost**					0.635
$200 or less	13	29.3 [16.1, 47.3]	28	29.7 [20.3, 41.2]	
$201-$400	11	35.9 [20.3, 55.2]	23	24.5 [16.2, 35.3]	
$401 or more	11	23.6 [12.1, 40.8]	28	32.8 [22.6, 44.9]	
No cost	5	11.2 [4.3, 26.4]	10	13.0 [6.7, 23.7]	

## Discussion

We estimate that approximately 2.9 million PMF owners owned 10.1 million PMFs at the end of 2024, roughly one half of which (4.9 million) were unserialized. Most PMFs were constructed using assembly kits or precursor parts (one fourth were 3D-printed or machined from raw materials), and most cost less than $400.

We asked PMF owners to tell us about their most recently acquired PMF. Among these, we found that those acquired within the past 24 months were more likely to have been serialized than those acquired more distally. Although we do not know where owners acquired their most recent PMF (e.g., from an FFL, private party, etc.), an increase in the proportion of serialized PMFs is broadly consistent the intended effect of ATF Final Rule 2021R-05 F. Our finding of increased serialization represents a weighted average of the effect of Final Rule 2021R-05 F on the PMFs it targets and the serialization of those PMFs not subject to the rule (i.e., 3D printed and milled firearms that are not transferred by a federally licensed dealer). As 3D printing technology becomes easier to access and more affordable, this trend could be reversed. As such, extending the Final Rule 2021R-05 F to include these currently unregulated PMFs would increase the fraction of PMFs serialized still further. Future studies specifically examining the impact and limitations of the 2022 ATF rule are needed to improve ongoing regulatory efforts related to PMFs.

### Limitations

Our findings should be considered in light of several limitations. First, we chose to ask respondents first about the total number of firearms they owned, and then how many of the firearms they owned were PMFs. To the extent that any respondents did not consider their PMFs firearms we may underestimate the total number of PMFs. Given that data are self-reported, our sample is subject to potential reporting bias. If this potential bias, on average, reflects respondents less likely to report firearm ownership than respondents who exaggerate ownership, it could lead to an underestimate of the PMF stock.

Additionally, although we aimed to assess the impact of 2022 ATF rule on serialization and background check at time of most recent PMF purchase, our sample size of PMF owners was not large enough to directly analyze the effect of this regulatory policy. Furthermore, we did not ask respondents to classify their most recently acquired PMF in a sufficiently granular way to allow us to examine how our aggregate statistics about serialization and background checks may have been affected by changes in types of PMFs acquired or whether they were obtained through a licensed firearms dealer. Future work on trends in serialization and background checking should better specify these aspects of the acquisitions than we did in the current study.

## Conclusion

Approximately half of the 10 million PMFs owned by US adults were not serialized, including one-third of those acquired by adults who acquired a PMF since 2022. Recent changes in how PMFs are regulated appear to be associated with an increased likelihood that when someone purchases a PMF, or constructs one themselves, their PMF will be serialized and that they will undergo a background check as part of any transaction.

## Data Availability

The data that support the findings of this study are not openly available due to reasons of sensitivity and are available from the corresponding author upon reasonable request.

## References

[1] California DOJ, Office of Gun Violence Prevention, Report. Oct 2024 https://oag.ca.gov/system/files/media/ogvp-report-ghost-guns.pdf

[2] Privately Made. Firearms | Bureau of Alcohol, Tobacco, Firearms and Explosives. https://www.atf.gov/firearms/privately-made-firearms

[3] Privately Made Firearms Updates and New Analysis. National Firearms Commerce and Trafficking Assessment. Vol, Four. https://www.atf.gov/firearms/docs/report/nfcta-volume-iv-part-v-%E2%80%93-pmf-updates-and-new-analysis/download

[4] Summary of Final Rule 2021R-05F | Bureau of Alcohol, Tobacco, Firearms and Explosives. Accessed April 9, 2025 https://www.atf.gov/rules-and-regulations/definition-frame-or-receiver/summary

[5] Ipsos Public Affairs. National Firearms Survey 2024; 2025.

[6] von Elm E, Altman DG, Egger M, et al. The Strengthening the Reporting of Observational Studies in Epidemiology (STROBE) statement: guidelines for reporting observational studies. Lancet. 2007;370(9596):1453-1457. 10.1016/S0140-6736(07)61602-X 18064739

[7] U.S. Census Bureau QuickFacts: United States https://www.census.gov/quickfacts/fact/table/US/PST0452201

